# Dr. Mossman, on the Effects of Digitalis in Consumption

**Published:** 1799-10

**Authors:** George Mossman

**Affiliations:** Bradford


### *To the Editors of the Medical and Phyfical Journal.*

GENTLEMEN,

BEFORE I detail the following cafes of *phthifis*, permit me to affure Dr. MACLEAN, of Sudbury, that I feel highly obliged by his friendly hint refpecting the neceffity of having the genuine leaves of *digitalis*, properly collected and prepared. I am well aware, that without the moft faftidious attention to this matter, every attempt to appreciate the real qualities of the plant will be unavailing; I have therefore very generally inquired into the nature of the powder which I have employed. The greateft part of the *fox-glove* which I have hitherto prefcribed has been collected and dried by a gentleman of this place, Mr. MAUD, who is fcrupuloufly exact in his preparations, and who is equally ambitious with myfelf to unfold the pofitive powers of *digitalis*. As foon as I read the extract from Dr. Maclean's letter inferted in the laft number of the *Journal*, I folicited Mr. Maud to give me in writing a brief hiftory of his mode of collecting the plant, and his fubfequent quent management of it.—The following is the reply I had from that gentleman:—

" W. Maud's reſpects attend Dr. Moſſman: in compliance with his requeſt, and in reply to his inquiries relating to *digitalis*, he has ſent the following ſhort ſtatement:—W. M. believes he can aſſure Dr. M. that the *digitalis* uſed in all the preſcriptions W. M. prepared, was of the moſt unexceptionable quality, and prepared as nearly as poſſible, in ſtrict conformity to the ſtatement below:

" The *digitalis* employed has always been collected from healthy, vigorous plants, growing on hilly, uncultivated, or at leaſt in ſoils not manured, and in ſituations moſt expoſed to the ſun. Thoſe plants which have acquired a darkiſh, browniſh caſt are preferred. They are gathered in autumn, after they have received all the advantages of the ſummer heats, without ſuſtaining any injury from froſt. The leaves thus collected are carefully ſeparated from the ſtalks, and the latter are cut away, ſo as only to leave room to tye them into ſmall bunches, which are hung to dry in a warm, airy kitchin. When dry it is *partially* powdered, and by this means the ſtalks and more fibrous parts of the leaves, which require more beating, are completely, or at leaſt pretty fully ſeparated. The finer part of the leaves, when ſufficiently powdered, is cloſely bottled for uſe."

The *digitalis* ſo collected and prepared is what I have chiefly uſed. I perfectly agree with Mr. Maud in the preference which he gives to the leaves of a dark colour, approaching to brown. My ideas alſo correſpond with his, in chooſing thoſe plants which grow in ſituations the moſt expoſed; for I am very well perſuaded, that thoſe herbs excel in flavour and quality which are collected from the moſt elevated grounds.—This opinion requires no evidence to eſtabliſh it but the moſt common obſervation; hence I am induced to believe, that a garden is the worſt of all ſituations (except marſhy grounds) for the cultivation of *digitalis*, and that the plant which I have been employing is much ſuperior to that recommended by Dr. Maclean.

If Dr. Maclean will recur to the hiſtory of my caſes, he will perceive that my *uſual* method of adminiſtering the *fox-glove* is, by the exhibition of *a grain four times a day*. In the caſe quoted by the Doctor, my patient was labouring under a very ſevere *active hæmorrhage*, and I deemed it important to his exiſtence to leſſen arterial action as ſpeedily as poſſible. The employment of the remedies ſpecified in the hiſtory of the caſe, was ſo efficient as to render a long continuance of them unneceſſary; I have no hesitation, heſitation, however, in affirming that the patient was decidedly under the influence of *digitalis;* for I could plainly perceive ſome of the moſt prominent features of its effects, ſo very juſtly delineated by the Doctor himſelf. Dr. Maclean's paper is now before me; and I think his remarks entitled to high conſideration, inaſmuch as they ſeem to be the fruit of an unbiaſſed judgment, enlightened by ample experience. My obſervations are ſtill ſo narrow, that at preſent I am totally unprepared to decide concluſively upon the virtues of *fox-glove;* I feel, however, the importance of the ſubject; and am determined to proſecute my inquiries till I can at leaſt ſatisfy my own mind.—I ſhall only now obſerve, that opinions reſpecting its power have already forced themſelves upon me, which have conſiderably leſſened the terrors which I entertained reſpecting the iſſue of *phthiſis,* and I feel irreſiſtibly impreſſed with the idea, that that diſeaſe will, at ſome future period, ceaſe to be the *opprobrium* of our art.

BRADFORD, I am, Gentlemen, your's reſpectfully,

*Sept.* 10th. 1799. GEORGE MOSSMAN.

*Caſes continued.*

CASE 6, *June* 3.—S. P. aged eighteen, has laboured, more or leſs, for two years paſt, under ſymptoms of *phthiſis pulmonalis,* accompanied with *anaſarca* of the lower extremities—ſhe dates her complaints from ſleeping in a damp bed.—She has had ſeveral opinions upon her caſe, and ſhe has taken a variety of medicines, without obtaining any permanent relief. I preſcribed for her a grain of the *digitalis* to be taken four times a day, with a cupful of a ſtrong cold infuſion of *chamomile flowers.*

*June* 20.—In a few days after ſhe began the uſe of her medicines, the *anaſarcous affection* began to leſſen;—it is now entirely removed. She has taken the *digitalis* regularly, but has occaſionally complained of a ſlight giddineſs, accompanied by a tendency to drowſineſs; the pulſe is reduced from 120 to 100 ſtrokes in the minute. Her difficulty of breathing, ſhe ſays, is conſiderably relieved, but her cough is as diſtreſſing as ever. I ordered her to take only *two grains* of the *digitalis* daily, but ſtrongly recommended punctuality in taking this quantity.

*Aug.* 29.—I heard no more of her till this day, when I called to inquire after her, and was agreeably ſurpriſed to find her in the bloom of health.—She informed me, that finding the diſtreſſing ſymptoms of her diſeaſe gradually abating, ſhe continued to take her medicines for ſeveral weeks, till ſhe found them no longer neceſſary.

CASE 7. *July* 6.—M. P. a married woman, aged forty-eight, about Chriſtmas mas laft, was feized with a bad cough, pain in her fide and dyfpnœa, for which fhe took no medicine fince the period above mentioned; all her complaints have much encreafed—fhe is extremely feeble and emaciated—her pulfe is 120, fmall and irregular—her cough diftreffes her much, more efpecially in the night. What fhe expectorates is of a frothy appearance, and, as fhe fays, of a faltifh tafte. She cannot lay upon either fide without experiencing a very uneafy fenfation, accompanied with inceffant coughing—her dyfpnœa is great—her legs have lately become *œdematous*—her tongue is partially ftreaked with a white fur—fhe has great thirft and no appetite—colliquative fweats and diarrhœa alternate with each other. *Milk, eggs, broths, jellies, &c.* were recommended for her diet—fhe was ordered a grain of the *digitalis* four times a day—fhe had *troches of liquorice and opium* for her cough, and fhe had a large *blifter* applied to the cheft.

*July* 12.—The *blifter* operated extremely well, and gave her much relief—her cough is better, and expectoration more free—fhe fleeps and eats better, and feels ftronger than fhe was a week ago—her pulfe is reduced to 104—her ancles only are a little *œdematous* at night—her medicines were continued.

*Sept.* 4.—The pulfe is 70—her appetite good—her cough, difficulty of breathing, and all her complaints, have difappeared.

CASE 8. *July* 14.—H. S. aged fourteen, about fix months ago loft a brother by *phthifis*.—She was deeply afflicted by this event, and from the period of her brother's death, fhe has appeared to decline; her appetite has gradually impaired, together with her flefh and ftrength, and fhe is now much emaciated.—Her tongue is furred; fhe complains of a pain in her left fide, more efpecially upon a full infpiration—fhe has a fhort tickling cough—fhe has no perceptible chills, but a palenefs and flufhing of her countenance are frequently obferved to fucceed each other—her pulfe is 125—bowels regular—urine high-coloured—fhe has occafionally partial fweats—her fleep is much difturbed—her fpirits are very irregular—fhe has never menftruated. A diet of *milk* was exclufively enjoined, and the ufe of the *fwing* four or five times a day. I directed her to have a grain of *digitalis* four times a day, with a couple of table-fpoonfuls of Dr. GRIFFITH's *anti-hectic preparation*, fo much recommended in cafes of *phthifis*.

*July* 23.—Her medicines, &c. have agreed with her well—her appetite is better—her pulfe 100—her tongue clean—the pain in her fide much abated—all her complaints are very confiderably leffened, and fhe feems to approach rapidly to a ftate of the moft complete health.

*Sept.* 4.—For several weeks past she has enjoyed the most perfect health.

Case 9. *Aug.* 22.—J. R. aged twenty-three, caught cold three weeks ago by being wet, and sitting in his moist cloaths—he was soon after seized with cold shiverings, pain in his side, difficulty of breathing, and every symptom of *pneumonia.* Till to-day he had not applied for medical aid, but had imprudently taken a variety of heating cordials, which had been recommended to him by his neighbours—he cannot lie on either side without experiencing a sense of suffocation; he breathes with extreme difficulty; his cough is incessant, and without any expectoration, except a sort of blue-coloured froth—his pulse is 120—extreme prostration of strength—thirst considerable, and frequent rigors. To the exclusion of every thing cordial, I ordered his diet to consist of *milk* and *eggs*, &c. I prescribed four grains of the *digitalis* daily, the use of the *troches* specified in a former case, and the application of a *blister* to the chest.

*Aug.* 25.—The *blister* operated well—dispnœa much relieved, and expectoration much more free—no thirst remaining—appetite good—the most remarkable phenomenon, however, is the astonishing diminution of vascular action—his pulse is reduced from 120 to 74. He seems to approach fast to a state of convalescence—he has a singular sort of feeling, which he describes as preceded by a faintness, and terminating in a tendency to sleep. As his cough is still troublesome, I ordered him a *mixture* to be taken occasionally, composed of *oxymel scillæ, syr. ex althea, tinct. opii. camphorat. et vin. ipecac.* The *digitalis* was continued.

*Sept.* 6.—He is well—a slight affection of his head, which assails him at a particular time of the day, is the only complaint he has. Upon the supposition that it is a *nervous head-ach,* I have ordered him the *bark.*

[To be continued.]

